# £10,000 for Portsmouth Hospital

**Published:** 1921-02-26

**Authors:** 


					<?.10,000 for Portsmouth Hospit^'
The Committee of the Portsmouth War -^l.
recently gave ?10,000 to the Royal Portsmouth -eJOf)ri^
This sum is half the total raised by the War a pof*
Committee. The hospital has still in hand ?2> ' $e
tion of the Mayor's Maintenance Fund; and, so ^ \>e
present year is concerned, the position is " e$iste<*
better, though the former deficit of ?6,000 sti
in December last.

				

